# Expanded Roles and Recommendations for Stakeholders to Successfully Reintegrate Modern Warriors and Mitigate Suicide Risk

**DOI:** 10.3389/fpsyg.2020.01907

**Published:** 2020-08-21

**Authors:** Joseph C. Geraci, Meaghan Mobbs, Emily R. Edwards, Bryan Doerries, Nicholas Armstrong, Robert Porcarelli, Elana Duffy, Colonel Michael Loos, Daniel Kilby, Josephine Juanamarga, Gilly Cantor, Loree Sutton, Yosef Sokol, Marianne Goodman

**Affiliations:** ^1^Transitioning Servicemember/Veteran and Suicide Prevention Center, VISN 2 Mental Illness Research, Education and Clinical Center, James J. Peters VA Medical Center, The Bronx, NY, United States; ^2^Resilience Center for Veterans and Families, Teachers College, Columbia University, New York City, NY, United States; ^3^Institute for Veterans and Military Families, Syracuse University, Syracuse, NY, United States; ^4^Theater of War Productions, New York City, NY, United States; ^5^Starbucks Coffee Company, Seattle, WA, United States; ^6^Pathfinder.vet, New York City, NY, United States; ^7^US Army G3/5/7 Staff, Washington, DC, United States; ^8^NYC Department of Veterans’ Services, New York City, NY, United States

**Keywords:** modern warrior, veteran, servicemember, reintegration stressors, suicide prevention

## Abstract

This article draws upon the legends of warriors from ancient Greece and other traditions to illuminate the journey of Modern Warriors (MWs) who have served in the United States military over the last century. It then turns to stakeholders that can assist current MWs in their reintegration to civilian life and mitigate suicide risk. Until this point, without an existing and coordinated local, federal, non-profit, and private system, rates of suicide for post-9/11 MWs after leaving the military have greatly increased, especially for young and women MWs. This is due in part to the military satisfying many of MWs’ needs by providing units, leaders, and a mission during the Departure and Initiation stages of the MW journey. However, as MWs exit the military and face the difficult task of reintegration, the absence of units, leaders, and mission leads to deteriorating psychological health and increasing suicide risk. Written primarily by post-9/11 MWs, this article proposes recommendations for stakeholders to better reintegrate MWs and mitigate suicide risk. The authors strive to develop a system that satisfies MWs’ reintegration needs and enables MWs to be well positioned to continue their next ‘mission’ – to serve and improve society.

## Introduction

Jung (1960) and other scholars described how universal themes, or archetypes, within the collective unconscious help explain how human behavior and characteristics are shared across time and cultures. The warrior archetype has been a powerful force in both ancient and modern times. This archetype was actualized as perseverance, courage, and aggressiveness for ancient Spartan warriors at Thermopylae in 480 B.C. and for modern warriors (MWs; i.e., military veterans and servicemembers) during World War II (Moore and Gillette, 1990). Currently, an average of 17 MWs in the United States die each day by suicide (US Department of Veterans Affairs, 2019a), and only limited evidence supports specific risk assessment methods or suicide prevention interventions for this population (US Department of Veterans Affairs, 2018b). Learning from previous warrior cultures may help elucidate current systematic shortcomings surrounding MW suicide prevention.

More than 4 million post-9/11 MWs have served in the US military since 2001 (US Department of Veterans Affairs, 2018e), comprising approximately 20% of the 19 million MWs in the United States (US Department of Veterans Affairs, 2019b; US Census, 2020). Combat exposure and other service-related stressors directly contribute to the onset of psychological disorders in this population (Booth-Kewley et al., 2013; Gradus et al., 2017). As more MWs continue to reintegrate to civilian life after military service, it is important to consider how contending with reintegration stressors impact MWs’ psychological wellbeing and risk for suicide (Geraci, 2018; Mobbs and Bonanno, 2018). At highest risk are young post-9/11 MWs (between the ages of 18–34); their rate of suicide has increased significantly more than any other age group, with rates doubling from approximately 22 suicide deaths per 100,000 population in 2006 to 44.5 suicide deaths per 100,000 population in 2017 (US Department of Veterans Affairs, 2019a).

These disturbing trends led President Donald Trump to sign two recent executive orders to curb MW suicide. Executive Order 13822 aimed to specifically support MWs by improving access to mental health care throughout the transition period (Executive Order, 2018). Similarly, Executive Order 13861 was issued in March 2019 to establish the ‘President’s Roadmap to Empower Veterans and End the National Tragedy of Suicide’ (PREVENTS) task force charged with focusing on community engagement strategies (Executive Order, 2019). PREVENTS attempts to apply early intervention and engage the broader community to reach MWs before they experience extensive mental health symptoms or an acute crisis. The order encourages federal government, academia, employers, members of faith-based and other community, non-governmental, and non-profit organizations and the veteran community to all work together to develop a strategy that will truly impact the current epidemic faced by MWs, especially the youngest transitioning MWs.

Most authors of this article are post-9/11 MWs, recently transitioned from the military and have become relatively successful in their respective civilian areas. They all faced, and continue to face, unique challenges during their ongoing reintegration to civilian life. Together, we write this article at a critical time, as national leaders and decision-makers reevaluate strategies for reintegrating MWs and mitigating suicide within this population. Recommendations made in this article are based on real-life experiences and an in-depth understanding of shortfalls in the current approach to MW reintegration and suicide prevention. The voices of post-9/11 MWs are rarely heard in academic literature or public policy debates related to these topics. This article attempts to integrate these voices to inform the PREVENTS task force and other national efforts to influence the next chapter of history with hopes for better outcomes for our fellow MWs.

We first apply Joseph Campbell’s Hero’s or Warrior’s Journey (Campbell, 2008) and Abraham Maslow’s theory of the Hierarchy of Needs (Maslow, 1987) to introduce and provide a framework for conceptualizing the reintegration challenges of MWs. Based on this conceptualization, we then provide recommendations (Table 1) for federal, local, private and non-profit organizations, and colleges to better assist MWs in their reintegration process and mitigate their suicide risk. Our intent is not to fully explain the challenges faced by MWs or to propose that the recommendations we will introduce are exhaustive. Rather, our goal is to move the dialog closer to a conceptual framework that can guide MWs, their families, and the entities responsible for their reintegration.

**TABLE 1:** Expanded roles and recommendations to better reintegrate modern warriors.

Department of Defense	– Increase the prioritization of MW reintegration in its vision and strategy to successfully reintegrate MWs and to help create recruiting ambassadors in local communities
	– Expand concept and resources for TAP to consist of ‘Phase One’ conducted within the DoD and ‘Phase Two’ conducted in MWs’ hometowns with DoD coordination
	– Similar to recruiters, position DoD resources *within* the local communities (like SFL Regional Outreach Teams)
	– Share local government best practices with other communities
Local Government	– Play the leading role in filling the organizational gap regarding MW reintegration by implementing a strategy that synchronizes resources, creates a spirit of collaboration, and addresses MW unique needs
	– Create a ‘no wrong door’ approach
	– Vet stakeholders that provide resources to MWs and hold them accountable for the quality of their services
Department of Veterans Affairs	– Provide best possible, MW-centered health services and benefits; attract and retain eligible MWs
	*Attract and Enroll:* – Increase VA involvement in DoD TAP programs; Provide liaisons to MWs to assist them in applying for VA benefits; Set up initial VA health appointment in their hometown
	– Play bigger role in MW reintegration by integrating VA reintegration efforts with local government strategies to support a ‘no wrong door’ approach
	– Evaluate the impact of the current VA motto upon enrolling women MWs
	*Retain:* – Standardize evaluation procedures for frontline staff regarding customer service
	– Provide local VA leadership authority over frontline staff and hold them accountable for customer service provided to MWs
	– Evaluate internal promotion policies, recruiting efforts, hiring initiatives, and scholarship programs to increase number of MW providers and leadership
	– Increase evidence-based Veteran culture competence training for all non-MW providers
Veteran Service Organizations	– Increase interoperability and collaboration with other stakeholders by participation in community– based digital platforms
	– Provide peer-to-peer support to MWs, such as Expiration Term of Service Sponsorship (Geraci et al., 2019)
Employers	– Chief Executive Officer (CEO) or leader must see the importance of and be genuinely committed to recruiting, hiring, retaining and advancing MWs
	– Develop a strategy that is resourced with a requisite team and funding
	– Create an energetic, employee-led affinity group that extends its hand to assimilate the newly hired MWs and spouses and can connect them to community resources
	– Expand evidence-based Veteran culture competence training
Colleges	– Leadership must see the importance of and be genuinely committed to recruiting, enrolling and graduating MWs
	– Develop a strategy that is resourced with a requisite team and funding
	– Create an energetic, student-led affinity group that extends its hand to assimilate the newly enrolled student MWs and can connect them to college and community resources
	– Expand evidence-based Veteran culture competence training
	– Expand MW-informed and led research that has the potential to mitigate MW suicide and facilitate successful reintegration

**FIGURE 1:**
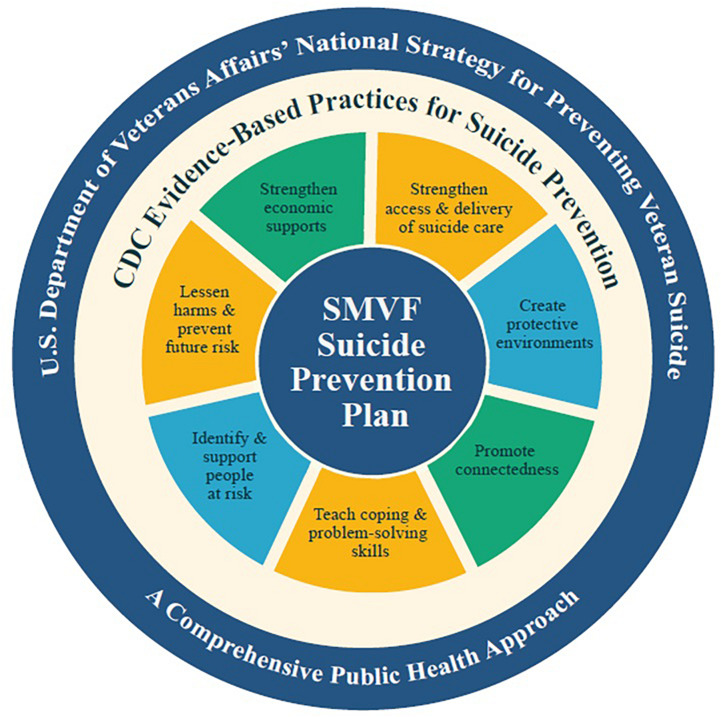
Service Member, Veteran and Family Member (SVMF) Suicide Prevention Model (Substance Abuse and Mental Health Services Administration [SAMHSA], 2020).

To help operationalize the recommendations presented in this article, the authors will introduce one promising initiative, the Expiration Term of Service (ETS) Sponsorship program (Geraci et al., 2019), that is already being implemented across the United States and is nested with the PREVENTS Task Force (2020) Roadmap recommendation to promote community-based models that are effectively implementing evidence-informed suicide prevention programs. ETS Sponsorship is such an evidence-informed program (Geraci et al., in press) that is a public-private partnership and synchronizes the efforts of the VA, the DOD, local governments, non-profit and community organizations, and corporations with all partners dedicated toward the goal of successfully reintegrating ***all*** MWs to their civilian post-military hometown, therefore helping to mitigate overall suicide risk. The program has also been implemented by some states as part of the Governor’s Challenge to Prevent Suicide Among Service Members, Veterans, and their Families run by the Substance Abuse and Mental Health Services Administration (SAMHSA) and the VA (Substance Abuse and Mental Health Services Administration [SAMHSA], 2020). The guiding framework for the challenge is the Service Member, Veteran and Family Member (SVMF) Suicide Prevention Model (Figure 1, Substance Abuse and Mental Health Services Administration [SAMHSA], 2020) that applies a public health approach to suicide prevention and integrates evidence-based practices for suicide prevention. The specific practices of the model that the ETS Sponsorship program emphasizes are ‘promoting connectedness, ‘strengthening economic supports’ and ‘identifying and supporting people at risk.’

## Warrior’s Journey

Though connected through the warrior archetype, each culture has developed its own rituals and rites of passage by which neophytes transform into warriors. Commonly referred to as the *warrior’s* or *hero’s journey*, this societal process creates individuals capable of fulfilling critical culture-specific roles. The ancient Greeks were one of the first cultures to document warrior legends to inform, shape, and inspire future warriors and communities. In the late 8th or early 7th century BC, Homer composed *The Iliad* and *The Odyssey*, epic poems recounting the exploits of heroes, such as Achilles, Ajax and Odysseus during and after the Trojan War. The stories of these Homeric heroes inspired Greek warriors for generations and continue to be relevant in understanding modern day warriors (Shay, 1994; Tick, 2005; Doerries, 2015).

Many authors have explored and linked ancient warrior legends and culture with modern-day equivalents, suggesting a common or shared warrior heritage and archetype. Joseph Campbell, for example, discovered that many common themes of hero and warrior myths exist across cultures, time, and space (Campbell, 2008). Likewise, Shay (1994) was struck by similarities between Vietnam war MWs’ experiences of losing comrades and Achilles’s struggles of profound grief and suicidal ideation after the combat death of his comrade in *The Iliad* (Homer and Butler, 2016). Shay’s most recent work, Odysseus in America (2002) used the Odyssey and Odysseus’ long journey home to explore the reintegration of combat warriors into civilian life through the lens of moral injury (MI) which can occur when warriors witness, fail to stop, or perpetuate immoral acts (Drescher et al., 2011).

The recommendations within the manuscript attempt to account for the heterogeneity of the MW experience and build on Shay’s work by acknowledging the unique experiences faced by MWs. Utilizing Campbell’s (2008) framework we categorize the warrior’s journey as occurring in three stages - Departure, Initiation, and Return. Throughout the following discussion of these stages, we will integrate the story of Odysseus to illustrate the qualities of each stage. This illustration highlights the courage and leadership required by MWs, as well as the challenges faced throughout the warrior’s journey.

### Departure

In the Departure stage, neophytes live in the ordinary world and are carried away from their everyday hut or castle to the threshold of adventure (Campbell, 2008). Odysseus was serving as King of Ithaca, tending to fields and living with his wife and son when he was called upon by Menelaus to assist in rescuing his wife, Helen, who had been abducted and taken to Troy. Odysseus was hesitant to leave his land and family. However, with time, he relented and even coaxed Achilles into joining him, Menelaus, Ajax, and others as they sailed to Troy at the beginning of the Trojan War (Statius and Lombardo, 2015).

Throughout history, military service has often been considered a test, and in some cultures, *the* test, of adulthood (Hall, 2011). Recently, most recruits have volunteered to join the military because of family tradition, a sense of duty, to acquire valuable skills and benefits (salary, medical, educational), to escape their previous lives, or to overcome a challenge and achieve a sense of purpose (Hall, 2011). During the Departure stage, the US military places great importance on senior warriors or ‘elders’ in assisting recruits. The US Army (Active) provides about 7,020 highly trained US Army Recruiting Command (USAREC) recruiters across the nation (US Army, 2014). To be eligible as a military recruiter, an individual must demonstrate at least 4 years of exemplary military service, be promoted to at least the rank of sergeant, be financially and emotionally stable, and pass a 6-week recruiter course (US Army, 2020a, c). These recruiters work in local communities, high schools, and community events to recruit, educate, guide, and enlist eligible civilians.

### Initiation

In the Initiation stage, neophytes must traverse the threshold into the ‘unknown world,’ accomplish numerous tasks, and overcome many threatening crises. The neophytes are usually confronted with their greatest fears and are brought to the brink of death in threatening encounters with hostile forces. With the assistance of helpers or elders, many prevail in this rite of passage, enabling them to undergo a warrior metamorphosis (Campbell, 2008).

Odysseus was known for his intellect and bravery during the Trojan War. With assistance from the goddess Athena, he developed the idea for the wooden Trojan horse and demonstrated his courage by leading Greek warriors in a covert operation to infiltrate the walls of Troy to defeat the Trojan army (Homer and Butler, 2016). Throughout the war, however, Odysseus also experienced the death of comrades and threats to his life. For example, Achilles, who Odysseus coaxed to leave his homeland and join at Troy, was ultimately killed, and Odysseus ended up retrieving Achilles’ body under heavy fighting with the Trojans (Homer and Butler, 2016).

In many ways, joining the military is the closest thing in our modern American culture to a traditional rite of passage. After enlisting, recruits’ transformation into MWs begins during the crucible of basic military training when their civilian identities are stripped away. One of the main intents of the training is to ensure recruits can persevere when faced with the horrors of battle (McGurk et al., 2006). This training enables recruits to access primitive and instinctual impulses to engage enemy combatants without hesitation while also instilling the self-discipline needed to control these impulses (Tick, 2005). Recruits learn to integrate values of loyalty to the group over the self, perseverance, and committing to a higher purpose with the willingness to make the ‘supreme sacrifice,’ death, to accomplish the mission (Tick, 2005; Redmond et al., 2015). This is the essence of the Soldier’s Creed, “*I will always place the mission first. I will never accept defeat…I will never leave a fallen comrade…I am a guardian of freedom and the American way of life*” (US Army, 2020e).

Forging this identity and inculcating these values is spearheaded by drill sergeants, who help recruits cross the threshold into the ‘unknown place’ and guide them through initiation. The Army uses a thorough process to select drill sergeants, who possess similar qualities and qualifications to recruiters (e.g., exemplary military service, promoted to at least sergeant, physically fit, emotionally stable, and passing a 9-week drill sergeant course; US Army, 2020b). With the assistance of drill sergeants, recruits complete their military training, pass their first rite of passage, and, thereby, earn the right to call themselves Soldiers, Sailors, Airmen, Marines or Coast Guardsmen.

The initiation stage continues with the MWs arriving at their first unit. Even prior to arriving, the military provides MWs with another helper or sponsor to help the MW integrate to their military installation and unit when they conduct a permanent change of station (PCS). For instance, the US Marine Corps has a very detailed and involved ‘PCS sponsorship’ program to reduce “stress and challenges associated with relocating” (US Navy, 2012, p. 2). The Marine Corps prioritizes this program by assigning responsibility for its successful execution to unit command teams. Sponsors of equal or higher rank are trained and optimally matched one-to-one with new MWs based on gender, marital status, and career field. Sponsors execute a wide variety of tasks, such as calling the incoming Marine to address their needs, arranging transportation, greeting incoming Marines and family members upon arrival, introducing them to key personnel, orienting them to the installation, and ensuring they have access to all required resources.

After integrating into their new units, the MWs receive tough, realistic, iterative, and dynamic training from unit leaders. Given the time spent between MWs and unit leaders, these leaders are probably the most important helpers during the MWs’ journey. Unit leaders have typically excelled in the military, graduated from military leadership development schools, and were selected by more senior military leaders for their position (US Army, 2020d). Many MWs within the US Army operate within a team of five, including four MWs and a team leader.

The importance placed on leader development and selection is underscored by the impact of leaders on the effectiveness of MWs and units (Bass and Riggio, 2006). In accordance with its objective for leadership, the Army prepares leaders to excel within the initiation phase so that they are smarter, more thoughtful, and innovative leaders of character, comfortable with the complexity found on modern battlefields (US Army, 2020d). By integrating ‘task-oriented’ leadership behaviors, new MWs’ leaders instill discipline and provide rigorous training that replicates situations found later in combat. Such training enables MWs to hone their military-specific skills and prepares them to face challenges during combat (Castro and McGurk, 2007; Geraci et al., 2011).

Many MWs rely heavily upon such training to accomplish their mission when facing combat exposure and possible death, particularly those involved in combat operations in Afghanistan and Iraq since 9/11. One study found that 83.3% of MWs deployed to these areas were attacked by insurgents and 82.9% knew someone injured or killed (Mental Health Advisory Team 6, 2009). From September 2001 through April 2020, over 5,400 MWs were killed in action, and over 52,950 MWs were wounded in action in Afghanistan and Iraq (US Department of Defense, 2020). Such combat exposure provides a second rite of passage and an “experience from which there is no return…the initiate is forged, through immersion in fire and blood, danger and threat, comradeship and fear, into something else” (Tick, 2005, p. 176)^1^.

Unfortunately, this rite of passage can have tragic effects on MWs’ mental health. Booth-Kewley et al. (2013) found a causal relationship between amount of combat exposure and later development of posttraumatic stress disorder (PTSD). PTSD prevalence rates for combat post-9/11 MWs are estimated between 4.5 to 19.9% (Magruder and Yeager, 2009; Bonanno et al., 2012), approximately two to four times higher than the general civilian population (Richardson et al., 2010). PTSD and number of combat deployments are also associated with increased risk for suicide attempt (Nock et al., 2014). Suicide was the second leading cause of death for MWs serving in the military on active duty from 2006 to 2018 (23.2% of all deaths; Mann and Fischer, 2019).

Research also suggests a potentially positive impact of leaders on safeguarding the MWs’ psychological wellbeing. U.S. Marines who rated their leaders as highly effective after a deployment to Afghanistan were about 50% less likely to develop a mental disorder than Marines who rated their leaders rated as ineffective (Booth-Kewley et al., 2013). The US Army realizes this connection between quality leadership and subordinates psychological wellbeing, noting that “leaders remain aware of the emotional toll that constant combat takes on their subordinates” and “provide emotional ‘shock absorbers’ for their subordinates” (US Army, 2006, section 7-12). In addition to training MWs around military tasks, leaders become these emotional ‘shock absorbers’ when they integrate effective ‘relational-oriented’ leadership behaviors, such as establishing positive interpersonal relationships with their subordinates and showing concern and respect to protect MWs’ psychological wellbeing and improve performance (Derue et al., 2011; Geraci et al., 2011).

It is important to note the unique challenges faced by women MWs whose initiation might be compounded by feeling forced to suppress their femininity to fit into a male-dominated military culture that values masculinity (Demers, 2013). As many as 54% of women MWs reported elevated emotional sensitivity as a result of having to tolerate gender discrimination (i.e., held to a higher standard compared to male MWs) and harassment (i.e., sexist jokes, crude remarks, and other forms of sexual harassment) on top of the stress of combat, and during reintegration (Lipari et al., 2008; Street et al., 2009; Demers, 2013; Gutierrez et al., 2013). Often these experiences leave women MWs with feelings of disillusionment and alienation from their male unit members and the civilian society at large (Demers, 2013). Women MWs are also at a higher risk of military sexual trauma, placing them at a significantly elevated risk for suicide compared to their civilian counterparts (Zarembo, 2015; Cuhalev, 2019).

### Return

In the Return stage, warriors must again pass through the threshold from the unknown world back into the ordinary world. Now transformed, they share the wisdom gained from their journey with others. The return is the final crisis in the warrior-journey, and many fail. It can be the most difficult aspect of the journey; upon returning to their everyday lives as civilians, warriors must contend with new and unwelcome stressors, including discomfiting queries, hard resentment, and misunderstanding (Campbell, 2008). If warriors receive the blessing and assistance of helpers or elders, they are more likely to successfully navigate the return and to utilize the wisdom, insights, and perspective gained during military service to bring about restoration of society (Campbell, 2008). However, if assistance is not provided, many warriors become lost between worlds.

According to Homer’s Odyssey, Odysseus faced 10 years of extensive challenges on his voyage back to his homeland, Ithaca. Odysseus and his warriors were taken prisoner by a Cyclops. His men became addicted to the narcotic lotus plant and lost their desire to return home. Odysseus and his men traveled to the underworld to face the dead and gain the advice of the blind prophet Tiresias. They escaped the deadly Sirens, fought monsters—Scylla and Charybdis—and weathered storms at sea that claimed the lives of most of Odysseus’ warriors and all of his ships (Homer and Butler, 2016). After Odysseus faced these challenges, he finally washed up on the island of Phaeacia, having lost all his men. King Alcinous, of Phaeacia, welcomed Odysseus into his home and, after hearing Odysseus’s story, offered to help him navigate the rest of the journey.

When Odysseus finally arrived home on the banks of Ithaca, Athena informed him of the many suitors living in his palace courting his wife, Penelope. This caused him to mistrust his wife and those within his place. Therefore, Athena disguised Odysseus as a beggar, enabling him to infiltrate the palace. Once inside, Odysseus removed his disguise and a skirmish ensued. When the suitors gained an advantage over Odysseus, he began to panic. At this critical moment, Athena appeared to Odysseus in the form of his old comrade, ‘Mentor.’ She helped restore Odysseus’ fighting spirit by urging him, “Come on my good fellow, stand by my side” (Homer and Butler, 2016, p. 614). As a result of Mentor/Athena’s support, Odysseus prevailed over the suitors, liberated his home, eventually reconnected with his wife, and reclaimed his throne.

Figuratively, Odysseus’ homecoming skirmish may represent the challenges faced by MWs in their attempt to return to civilian life. MWs contend with many reintegration stressors threatening their psychological wellbeing (Geraci, 2018; Mobbs and Bonanno, 2018). Recent studies suggest 44% of MWs experience great difficulty as they reintegrate into civilian life, including difficulties in employment, professional relationships, family, friend, and broader relationships, adapting to the schedule of civilian life, and legal involvement (Morin, 2011; Castro and Kintzle, 2014). Many returning MWs also exhibit high rates of substance use disorder (Teeters et al., 2017). It is likely that the current situation that many MWs face as they transition out of the military, due to the Coronavirus Disease 2019 pandemic, will further exacerbate their reintegration difficulties.

Adding to the complexity of the return home, the MWs who experienced morally injurious events or acts of perpetration and betrayal (Litz et al., 2009) during their time in service exhibit more severe, treatment resistant symptoms of PTSD (Steenkamp et al., 2015). These MWs report more pervasive complex emotions such as guilt, shame, anger, outrage, and frustration (Stein et al., 2012) which only further propel them to engage in self-handicapping and self-harming behaviors (Litz et al., 2009). Like Odysseus’ initial mistrust toward his wife, MWs with deep moral injuries are highly suspicious of others, including those closest and dearest to them (Shay, 2002). This can make it difficult for them to reconstruct their identity in civilian life and assume civilian values and practices that may not align with their MW identity. Without tangible support from helpers during reintegration, such as provided to Odysseus by King Alcinous and Athena, many MWs fail in their efforts to overcome these difficulties.

Moreover, research suggests the experience of reintegration stressors is associated with various deleterious outcomes. For example, these stressors are not only highly correlated with psychological problems (i.e., PTSD, depression and alcohol dependence), but lead to the 5.4 times higher odds of suicidal ideation found among MWs experiencing the highest number of reintegration stressors (Kline et al., 2011). As may be expected, the increased suicide ideation/attempts are not limited to those with a prior suicide attempt history; most MWs who attempt suicide make their first attempt after military separation (Villatte et al., 2015).

Again, here there are unique challenges for women MWs as they re-forge their womanhood in a new cultural context (Grimell, 2017) and may return to civilian life with social and physical health problems (Burkhart and Hogan, 2015). Even as women make up the largest growing segment of the MW population (Burkhart and Hogan, 2015) and the fastest growing segment of eligible VA health care users (Meehan, 2006), providers may fail to understand the unique needs of these women MWs and risk exacerbating trauma experienced during their military service through secondary victimization (Campbell and Raja, 2005; Zinzow et al., 2007; Burkhart and Hogan, 2015). During the return to civilian life, many women MWs avoid the conflict of experiencing the negative aspects of their time in uniform (i.e., MST, feelings of betrayal) while simultaneously feeling a deep pride in their service (Burkhart and Hogan, 2015).

## Reintegration Needs

In addition to the framework provided by Odyssey’s journey, Maslow’s theory of the Hierarchy of Needs (1987) may be used to understand the difficulties MWs face during reintegration. Maslow (1987) stated that human behavior and motivation are determined in large part by an individual’s ability to satisfy a series of needs arranged in a hierarchy. Based on Maslow’s theory, if reintegrating MWs are unable to satisfy needs at lower levels in the hierarchy, they will have less motivation to satisfy higher level needs required to complete their warrior journey.

Maslow (1987) considers physiological needs (food, water, shelter, transportation, etc.) to be the most prepotent; if unsatisfied, physiological needs become dominant and preclude other higher-level needs. Love and belongingness are the next level in the hierarchy and involve the desire to establish relationships with friends, significant others, family members and the community. The next level is self-esteem. Most people feel a need for a stable and high self-evaluation of themselves and the respect or esteem from others. Lastly, the final need, self-actualization, refers to the desire for self-fulfillment or to actualize an individual’s potential and is related to the ability to identify and fulfill their meaning in life.

### Physiological Needs

The military satisfies most physiological needs of MWs. This includes a salary to purchase or pay for transportation, food provided by dining facilities, a clothing allowance for uniforms sold on the military installation, housing in the barracks or on the installation, and medical care. Physiological needs continue to be met when MWs deploy to war. Consequently, many MWs experience a shock when they transition from the more collectivist community of the military where these needs are met to the more individualist one found in civilian society (US Department of Veterans Affairs, 2017). Though not nearly as extensive as provided within the military, AmericaServes is a national service network across the nation that attempts to streamline services available to reintegrating MWs in the post-military hometown. Specific to the importance of physiological needs for reintegrating MWs, the most requested service that MWs request from AmericaServes is related to housing (23% of the 77,671 total requests; Syracuse University IVMF, 2020). This is important to identify since over 9% (or 40,056) of all adults experiencing homelessness in the United States are MWs with two-thirds staying in shelters or transitional housing programs, while the other one-third being unsheltered, living in cars, in encampments, or on the streets (US Interagency Council on Homelessness, 2018).

These physiological needs overlap with the SMVF Suicide Prevention Model’s evidence-based practice of ‘strengthening economic supports’ for those at risk for suicide. This is especially important as economic strain, such as job loss, reduced income, difficulty covering medical, food, and housing expenses may increase an individual’s risk for suicide (Stack and Wasserman, 2007). So, any MW suicide prevention initiatives should help to ensure the physiological needs of MWs are met. According to Maslow’s theory of the hierarchy of needs, if MWs are not able to satisfy physiological needs, like housing, then it is highly unlikely that they will satisfy higher-level needs.

### Love and Belongingness Need

Modern warriors’ needs for love and belonging are fulfilled in their time in the military as they fight for and defend each other and, in the process, often experience the deepest love of their lives (Junger, 2016). Lieutenant General (retired) Hal Moore captured the essence of this love after his experience as the battalion commander for 1st Battalion, 7th Cavalry during the Vietnam War. During the Battle of Ia Drang, his unit was encircled by a numerously superior enemy; he later wrote, “We discovered in that depressing, hellish place, where death was our constant companion, that we loved each other” (Moore and Galloway, 1992, prologue). This social support has been widely studied and appears to protect against the development of PTSD (Haglund et al., 2007; Pietrzak et al., 2009). These ties are usually severed when MWs depart the military.

Most MWs likely perceive a deficit in support from helpers in the Return stage as compared to during the Departure and Initiation stages. This disparity is particularly salient within the US Army when comparing resources dedicated to recruiting new MWs to resources dedicated to reintegration. Throughout their journey, the support received from helpers is critical. Nevertheless, at a time when tangible support is needed, such as received by Odysseus from Athena/Mentor, there are no US Army helpers - recruiters, drill sergeants, or unit leaders. Prior to the ETS Sponsorship program, there were no sponsors like the PCS sponsors received when MWs move from one military installation to another. There are no military organizations, similar in size and scope to the USAREC, to assist with reintegration.

Additionally, when MWs return home, there are far fewer individuals, supervisors, family members and colleagues with whom they can relate. Unfortunately, this military-civilian divide may further exacerbate feelings of isolation and inability to fulfill love and belonging needs (Carter et al., 2017). This is especially true in recent years; during the height of conflicts in World War II, the Korean War, and the Vietnam War, a much larger percentage of the US population served in the active-duty US Army (5.9, 1.0, and 0.80%, respectively) compared to the 0.20% of the US population that serve in the active-duty US Army after 9/11 (US Department of Defense, 2018; US Census, 2020). In the current era, 84% of MWs believe that the civilian public does not understand their experiences or problems, and 71% of civilians acknowledge a lack of understanding, with half saying the recent wars in Iraq and Afghanistan had little impact upon their lives (Pew Research Center, 2011). Therefore, it is not surprising that MWs report feeling closer to their military comrades than to their civilian counterparts after exiting the military (Koenig et al., 2014).

Love and belongingness needs overlap with the SMVF Suicide Prevention Model’s evidence-based practice of ‘promoting connectedness,’ which helps to increase the support, love and belongingness that individuals feel from others. Such connectedness has been an important element of suicide prevention dating back to 1897 when scientists identified a lack of connectedness, love or social support as among the chief causes of suicidality (Durkheim, 1897). Joiner’s (2005) Interpersonal−Psychological Theory of Suicidal Behavior also supports the importance of promoting connectedness and theorizes that ‘thwarted belongingness’ (or a lack of connectedness, love or belonging) is a crucial ingredient for suicide. An additional benefit to this connectedness is that it will place trusted individuals, especially when they are trained, close to at-risk populations that can better assist in ‘identifying and supporting people at-risk,’ which is another evidence-based practice.

### Self-Esteem Need

Many MWs fulfill their esteem needs in the military by developing military-specific skills during basic and advanced training, which are further honed during real-world missions. Their lives and those of their comrades depended upon the MW’s proficiency in these skills. As MWs return to civilian society, many experience a reduction in esteem, because some of the skills relied on in the military are no longer directly beneficial. Like Odysseus, they may feel a desire to disguise their true identity as a MW due to thinking employers perceive them as dangerous or having a psychological disorder (Carter et al., 2017). Difficulties translating their skills may partially explain the challenges MWs experience with civilian employment (Carter et al., 2017). Compared to their non-MW counterparts, MWs tend to experience greater unemployment rates, particularly those who are younger, women, and less educated (US Department of Labor, 2020).

Maintaining employment after a position is secured is also a noticeable challenge for many MWs. Nearly half of all post-9/11 MWs leave their first civilian job during the 1st year of employment due to issues of esteem, lack of opportunity to apply previously learned skills and abilities, benefits/pay, and meaningfulness of the work (Maury et al., 2016).

Related to self-esteem, a growing body of empirical literature suggests that self- compassion may be important for understanding a range of mental health problems (Hiraoka et al., 2015) to include PTSD and moral injury. Conceptualized as three interacting components that emerge when experiencing emotional suffering: self-kindness (vs. self-judgment), a sense of common humanity (vs. isolation), and mindful awareness (vs. overidentification with suffering) (Neff, 2003a, b), recent research suggests it is possible residual symptoms such as shame or guilt, sometimes reported upon completion of an empirically supported treatment for PTSD (Lee et al., 2001), may be effectively alleviated by interventions designed to increase self-compassion. Prior research also suggests that increased self-compassion may be associated with improvements in life satisfaction and social connectedness (Neff and Germer, 2013) and relationship functioning (Neff and Beretvas, 2012; Yarnell and Neff, 2013). Therefore, self-compassion, in conjunction with broader self-esteem, may have implications for reintegration.

### Self-Actualization

Many MWs derived a sense of fulfillment from applying military skills to accomplish a military mission. Although MWs can feel constantly “on edge” during deployment, many consider it the highlight of their life and something they would gladly repeat (Castro et al., 2015). MWs faced and prevailed over death through a commitment - to the military, their unit, their unit’s mission, and its members. This accomplishment often imbued them with a sense of purpose; they believed they were making the world a better and safer place (Martin and McClure, 2000; Hall, 2008). Exposure to these situations led MWs to be changed forever (Campsie et al., 2006). However, this empowering transformation has an ironic aftereffect; as MWs return to civilian life, many feel unfulfilled, empty, and without purpose (Castro et al., 2015). Their personal and social identities, anchored within military culture, are lost. Mobbs and Bonanno (2018) suggest MWs may even experience grief-like symptoms during their reintegration where they mourn the loss of their military identity. Correspondingly, lack of fulfillment and meaning are related to symptoms of depression (Blackburn and Owens, 2015) and PTSD severity after military service (Owens et al., 2009). To protect against this loss of self-worth, it is critical for MWs to have opportunities to restore their sense of self-fulfillment and meaning.

## Way Ahead

A few important themes emerge when applying the warrior journey and Maslow’s Hierarchy of Needs to MWs. First, a “disease-based” model used alone to screen, diagnose, and treat specific psychological disorders that increase the risk for suicide is inadequate to address the epidemic of MW suicide. Given the MW mindset and ethos (i.e., being strong and reliable) instilled early in basic training and reinforced throughout military service, the resulting and pervasive stigma against receiving mental health care challenge the ability of a disease-based model to accurately and successfully screen, refer, engage, and retain MWs in needed evidence-based care.

Regarding screening, one study assessed the suicide screens provided to MWs in VA care (Louzon et al., 2016) and found that 71.6% of the 310 MWs who committed suicide within a year after receiving a suicide screen denied having any suicide ideation during the screen. So, the MWs screened negative but still later committed suicide. Further, many MWs who do screen positive for a mental health disorder or suicide risk will not follow through on referrals for mental health care. For instance, only 34% of MWs who screened positive for PTSD after a deployment to Afghanistan sought treatment within their 1st year of reintegration despite being eligible for care (Interian et al., 2012). Unfortunately, MWs who need but are not seeking mental health care from the VA face higher risk of suicide (US Department of Veterans Affairs, 2018f); from 2016 to 2017, the rate of suicide among MWs in recent VA care increased by 1.3 percent compared to increasing by 11.8 percent among MWs who did not use VA care (US Department of Veterans Affairs, 2019a). Further, many MWs who do seek treatment drop out of care before attending a sufficient number of sessions (Hoge et al., 2014; Steenkamp et al., 2015; Szafranski et al., 2017). For example, only 3.3 percent of MWs with a new-onset diagnosis of depression, anxiety or PTSD completed eight or more psychotherapy sessions at the VA (Mott et al., 2014), which is the minimum length of traditional, evidence-based psychotherapies (Beck et al., 1979; Foa et al., 2007). And for those that stay in treatment, approximately two-thirds of them still retain their mental health diagnosis after completing an EBT (Steenkamp et al., 2015).

Relatedly, currently available interventions for returning MWs have focused narrowly on extreme psychopathology, and typically only on PTSD (Mobbs and Bonanno, 2018). The resulting trauma-oriented focus and VA prioritization of cognitive processing therapy (CPT) and prolonged exposure (PE) (Friedman, 2006; Institute of Medicine, 2007; US Department of Veterans Affairs, and US Department of Defense, 2010; Yehuda and Hoge, 2016a, b) can obscure events that provoke shame and guilt but are unrelated to hyperarousal (Litz et al., 2009).

So, in addition to a disease model, we place increased emphasis on a “MW-based” model (Geraci et al., 2020b); nested within military and warrior cultures, an MW-based model can be applied to ***all*** MWs, families, and communities and may lead to better outcomes, including a decreased number of MW suicides. This model acknowledges that reintegration is intrinsically challenging, the needs of each MW are unique, many MWs are under-prepared for the reintegration, and unaddressed reintegration stressors can increase suicide risk. Some MWs may require extensive assistance meeting physiological and medical needs, while others may require more support building self-esteem in the civilian workplace. The MW-based model identifies the potential of ***all*** MWs to satisfy the highest-level need of self-actualization and fulfillment, characterized by the ability to integrate wisdom obtained from the military for the betterment of their civilian communities. Such an approach is consistent with the VA’s recent “National Strategy for Preventing Veteran Suicide” (US Department of Veterans Affairs, 2018d), which calls for an increased emphasis on a comprehensive public health and universal approach to suicide prevention and focuses on the entire population of reintegrating MWs.

Second, all MWs require helpers or elders alongside them throughout the reintegration process to offer advice and serve as amulets protecting against evil, danger, and disease (Campbell, 2008). Tick (2005) describes the importance of elders for warriors in many Native American cultures: training warriors, guiding them through tests and battle, and conducting rituals and providing assistance as warriors are reintegrated into the tribe. The elders accessible to post-9/11 MWs in the Departure and Initiation Stages are well-trained and selected. However, these elders are noticeably absent during the Return Stage, where the aid of elders or helpers may be most vital for MWs’ well-being as they strive to accomplish love and belongingness needs.

Third, elders and MWs should operate under the support of higher organizations that allocate and synchronize resources to ensure success. For instance, the US Army allocates resources, consistent with its vision of five critical objectives (i.e., manning, organizing, training, equipping and leading) to ensure MW success during the Departure and Initiation Stages. However, MW reintegration back into civilian communities is noticeably absent from the Army vision and strategy (US Army, 2018). In fact, a cynical observer may wonder if the US Army perceives reintegration resources as incentive for attrition and therefore counterproductive to its stated goal to “maximize unit manning…and retain the most qualified (MWs)” (US Army, 2018, p. 5). Regardless of cause, insufficient resources in MWs’ hometowns create an organizational gap encumbering MWs’ safe and effective reintegration.

With these themes in mind, we propose expanded roles and recommendations for stakeholders to better assist MWs with reintegration. We highlight examples from the ETS Sponsorship program, as applicable. ETS Sponsorship was originally established in 2013 based on recommendations presented by the DOD Defense Center of Excellence for Psychological Health and TBI in its Best Practices Identified for Peer Support Programs report (Department of Defense, 2011).

ETS Sponsorship helps sponsorship help to synchronize the efforts of the many stakeholders referenced below by enrolling MWs approximately 6 months prior to them exiting the military, matching them with volunteer and certified sponsors that are in the post-military hometowns, and by connecting them to community networks and services (VA and non-VA) available in these post-military hometowns. A randomized controlled trial study evaluating the ETS Sponsorship was conducted in New York City with 203 recently integrated MWs with positive results (Geraci et al., in press). With a goal of providing this one-on-one program for all transitioning MWs, the ETS Sponsorship program has manualized (Geraci et al., 2020a) its certification process to ensure that all of its sponsors attend training sessions that certify their skills related to three of the evidence-based practices identified within the SMVF Suicide Prevention Model- ‘promoting connectedness, ‘strengthening economic supports’ and ‘identifying and supporting people at risk.’

The initial results of efficacy of the ETS Sponsorship program influenced certain leaders within the VA to take additional steps to integrate ETS Sponsorship into their respective regions. For example, the VA leadership in Texas is funding the operational expansion of ETS Sponsorship across the state of Texas from 2020 to 2023, as well as the funds to conduct a thorough program evaluation of the expansion. This evaluation will enable the VA to determine to what extent the program is able to strengthen economic supports, reduce reintegration stressors, promote connectedness and reduce suicide risk for reintegrating MWs. This work in Texas in noteworthy as almost 20% (or approximately 9,500) of all Soldiers existing the US Army-active duty every year will establish their post-military hometowns within Texas (US Army, 2017).

Overall, we strive for MW reintegration to be a process and an outcome after which MW needs (physiological, love and belongingness, esteem, and self-actualization) are satisfied. Through reintegration, MWs should experience improvements in physical and psychological well-being and become well positioned to continue their next ‘mission’ of serving and improving society with a newly forged MW identity, wisdom, and sense of purpose (Elnitsky et al., 2017; US Department of Veterans Affairs, 2017; Geraci et al., 2020b). See Table 1 for a summary of these expanded roles and recommendations.

### Department of Defense

To improve MW reintegration under a comprehensive and public health approach to suicide prevention, we recommend the DoD play a greater role in the Return stage to address problems “upstream of the separation date” (US Department of Veterans Affairs, 2017, p. 11). Service branches should realize and identify the importance of their role in MW reintegration and allocate resources more on par with recruitment and training efforts.

To maintain support to MWs transitioning out of the military, Congress passed the Veterans Opportunity to Work (VOW) Act in 2011 (Public law 112-56, 2011). The VOW Act mandates MWs exiting out of the military to complete a transition assistance program (TAP). In line with this mandate, US Army revamped its Soldier for Life- Transition Assistance Program (SFL-TAP) and attempts to prepare MWs for a new career by having them participate in approximately 1 week of informational classes and workshops. MWs must receive an exit counseling session with a civilian transition services specialist (TSS) or counselor on their military installation (Public law 112-56, 2011; US Army, 2016a). However, as most MWs tend to reintegrate in a location not near their last military installation, it remains unclear to what extent these services adequately assist MWs with reintegration needs.

A separate entity from SFL-TAP, US Army Soldier for Life (SFL) has the mission to network with the community at large to shape education, employment, and health policies, programs and/or services on behalf of our Soldiers, Army Veterans and their Families. It helps to accomplish this mission by attempting to connect MWs with local services and resources through its four Regional Outreach Teams (Northeast/Europe, South, Midwest, and West/Pacific), each consisting of one officer and one master sergeant (US Army, 2016a). But since 62,872 enlisted MWs exited the US Army (Active) in 2016 (US Army, 2017), this means that there were approximately *7,859 enlisted MWs per each of the SFL outreach personnel* in the Return stage (see Table 2). With a similar number of Army recruits entering the Army each year as exiting, this lopsided ratio is in stark contrast to the *9.74 Army recruits per each of the Army (Active) recruiters* working in 982 Army recruiting stations nation-wide in the Departure stage (US Army, 2014).

**TABLE 2:** Ratio of US army ‘helpers’ in modern warriors’ hometowns (departure vs. return).

	Number of Enlisted MWs	Number of Helpers	Ratio (MW/Helper)
Departure	68,354 enlisted into US Army (Active)^1^	7,020 US Army (Active) Recruiters^1^	9.74/1
Return	62,872 exited the US Army (Active)^2^	8 US Army (Active) Regional Outreach Team personnel^3^	7,859/1

*^1^US Army (2014), ^2^US Army (2017), ^3^US Army (2016a).*

One option could be an expansion of TAP so that DoD’s efforts to meet VOW Act of 2011 requirements are considered ‘Phase One’ of transition for MWs prior to exiting the military. The DoD could then coordinate with agencies within the MWs’ hometowns to ensure that MWs conduct ‘Phase Two’ with these agencies (discussed below). As part of Phase Two, reviewing DoD TAP files and in-person meetings with MWs could help local agencies understand the unique needs of each MW and connect them with services in the local community. Local agencies could then advocate for MWs and serve as a ‘helper’ by providing tangible support to reintegrating MWs. These Phase Two efforts could help address problems that have “severely hamstrung” the ability of community entities to assist in MW reintegration (particularly problems around having access to MWs and understanding their needs; US Department of Veterans Affairs, 2017, p. 20). At the national level, allocating greater resources to DoD organizations (e.g., SFL Regional Outreach Teams) and placing these organization within their respective regions, rather than being located near the Pentagon, could enable the DoD to coordinate an effective implementation of Phase Two with local agencies and enable DoD to better disseminate best practices across the nation.

Related to ETS Sponsorship, the outreach team in the SFL South region has already been coordinating with garrison commands and SFL-TAP leadership on Army bases around the world to facilitate the enrollment of MWs still in the military, VA partners in Texas, and Texas governmental offices to facilitate the expansion of the ETS Sponsorship program across the state of Texas. An additional office within SFL, the Army Retirement Services, has also partnered with ETS Sponsorship to help recruit Retired Army Soldiers that have already exited the Army and successfully reintegrated into their post-military hometowns to serve as ETS Sponsors (US Army, 2020, June). To highlight the potential for future growth and the available pool of possible ETS Sponsorship candidates, there are 97,206 Retired Army Soldiers that live within the state of Texas (US Department of Defense, 2019). While not a requirement to be a MW to be a sponsor, the program gives Retired Soldiers the opportunity to bring to fruition the essence of the term ‘Soldier for Life’ and enables them to continue to serve the Army and their communities through helping the MWs who are soon to reintegrate into the very same post-military hometowns. Successful reintegration of MWs into these hometowns could also help to create ambassadors for potential Army recruits (Dickstein, 2018), which in turn, would reduce the burden of military recruiters.

### Department of Veterans Affairs

The VA is tasked with enrolling eligible MWs and providing the best possible, MW-centered health services and benefits to all eligible MWs. While not all MWs utilize the VA, its central role in MW medical and mental health care makes it a critical component of any successful system supporting MW reintegration. However, the VA reported that only 26.1% of post-9/11 MWs are enrolled in and use care provided by the VA’s Health Administration (VHA), excluding Vet Center use (US Department of Veterans Affairs, 2018e). The VA has made admirable efforts to increase utilization and eligibility of post-9/11 MWs, particularly those who served in combat and those that experienced military sexual trauma. This is important, as 58% of MWs who seek VA health care screen positive for a mental health diagnosis (Committee to Evaluate the Department of Veterans Affairs Mental Health Services, 2018). But additional improvements are needed in the areas of attracting and retaining eligible MWs. Related to suicide prevention and the importance of attracting and enrolling eligible MWs with the VA, research shows that over two-thirds of MWs who have died by suicide did not recently receive care from the US Department of Veterans Affairs (2018f).

#### Attracting and Enrolling Eligible MWs

To attract more eligible MWs and implement a universal approach to suicide prevention, the VA could improve efforts prior to MWs exiting the military. The VA has recently implemented some initiatives toward this end. For example, it now provides VA Liaisons at 21 military medical treatment facilities (MTF) for MWs struggling with medical and mental health needs (Miller, 2019). Committee to Evaluate the Department of Veterans Affairs Mental Health Services (2018) also offered to increase VA involvement in the DoD TAP programs by providing liaisons to all individual MWs, not just those currently suffering from mental and medical issues in the military, to assist them in understanding and applying for VA benefits and to assist eligible MW to set up an initial VA health appointment in their post-military hometown. Because 42% of MWs in need of VA care in their local community, who have not sought it, report being uninformed of the VA benefits application process (Committee to Evaluate the Department of Veterans Affairs Mental Health Services, 2018), such improvements could potentially address one of the main reasons for MWs underutilization of VA healthcare services.

Further, it is important for the VA to continue placing special emphasis on attracting women MWs and their unique needs. Women have heroically served our nation since its inception, demonstrated by the courage of Margaret Corbin when she was wounded while firing a cannon against British forces during the American Revolution (Berkin, 2006). However, the role of women in the military has been limited until more recent governmental actions. Only in 1967, when President Johnson signed Public Law 90-130, could women be promoted to general and flag ranks and the 2% ceiling on the number of women allowed to serve in the military was lifted (Public law 90-130, 1967). As a result, the percentage of women currently serving in the US Army (active) is 15% and growing (US Army, 2016b). Additionally, in 2015, Defense Secretary Ash Carter announced that all military occupations, including US Army Rangers and Green Berets, Navy SEALs, Marine Corps infantry, and Air Force parajumpers, would be open to women without exception, thus increasing the percentage of women being exposed to higher levels of direct combat (US Department of Defense, 2015).

The VA has attempted to reduce healthcare barriers for women MWs over the last decade, with the number of women MWs using VA health care nearly doubling to more than 500,000 (US Department of Veterans Affairs, 2015). However, recent estimates suggest the suicide rate for women MWs has increased to about 2.2 times that of non-MW women suicide rates (US Department of Veterans Affairs, 2019a). While the impact of expanded combat roles upon the psychological health of women is still unknown, it is important for the VA to attract and enroll more women MWs prior to their separation from the military and to be responsive to the unique needs of women MWs. As one example, given that women MWs can now serve within every combat position in the military, the VA could evaluate if making its current VA motto more gender-inclusive would attract more women MWs. Its current motto is taken from President Abraham Lincoln’s second inaugural address- “To care for him who shall have borne the battle and for his widow, and his orphan” (Wentling, 2018).

To attract more eligible MWs after they have exited the military, the VA has recently launched its Solid Start program with the VA calling every newly separated MW from the military three times during their 1st year of separation to ensure they are informed of eligible VA benefits (e.g., VA home loans, health care, employment opportunities, and mental health support; US Department of Veterans Affairs, 2020b). Such initiatives can help ensure MWs are familiar with and connected to VA programs that can serve as optimal entry-points into the VA, thereby helping to accomplish Phase Two needs. The VA could also play a bigger role in partnering with local communities to improve MW reintegration and support a ‘no wrong door’ approach. With slight modifications to VA policies and procedures, there are VA programs well positioned to enable the VA to play this bigger role in MW reintegration and connecting MWs to local community services. These local services could also serve as conduits to the VA for reintegrating MWs. An infrastructure already exists through the VA’s Community Veterans Engagement Boards (CVEBs) with a presence in over 170 cities across the United States that enable MWs, family members, MW advocates, community service providers, and stakeholders to have a collective voice in identifying their community goals and working to resolve gaps in service at the local level (US Department of Veterans Affairs, 2020c, April 2020).

For example, there are over 300 VA Readjustment Counseling Service/Vet Centers, staffed by dedicated mental health professionals, across the United States that offer a broad range of readjustment counseling services, in addition to providing information on VA benefits, and referrals to local VA and community resources (Botero et al., 2020). Additionally, every VA Medical Center has a Transition and Care Management Team specifically focused on welcoming eligible post-9/11 MWs, coordinating MW care activities, and aiding MWs as they navigate their way through the VA system (US Department of Veterans Affairs, 2020d). The Veterans Integration to Academic Leadership (VITAL) initiative also places VA mental health providers on college campuses in over 25 cities across the nation and facilitates MWs seeking student MW-specific care directly on their campus from the VA (US Department of Veterans Affairs, 2020c). This initiative is especially promising as education is one of the most utilized VA benefits, with 946,829 beneficiaries using VA educational benefits in 2017 (US Department of Veterans Affairs, 2018a).

Currently, however, there is no national strategy to integrate such innovative VA reintegration programs to connect them to all MWs prior to them exiting the military and to enable these programs to connect MWs to other local community services that operate outside of the VA. Though, the ETS Sponsorship program is actively working closely with important VA programs and initiatives to help ease the transition of reintegrating MWs into the VA and their local communities. Within Texas, the VA has hired clinical social workers to serve as ‘VA ETS Sponsorship Coordinators’ who work across the state to facilitate coordination with VSOs and best support both the MWs within the program and the hundreds of volunteer ETS Sponsors serving these MWs in Texas cities.

#### Retaining Eligible MWs

After the initiatives mentioned above attract and enroll MWs, it is critical for the VA to focus on retaining MWs in needed services. Committee to Evaluate the Department of Veterans Affairs Mental Health Services (2018) outlined recommendations to address issues of VA mental health services provided to post-9/11 MWs, such as the limited effectiveness and dropouts. One improvement we would like to highlight applies to customer service. They found that more than half (54%) of the MWs in their representative study reported having had a bad experience with the VA with more than three-quarters of all MWs indicating that better customer service (77%) is an important change. Also, MWs at 21 different VA sites complained to the committee about poor interactions with frontline staff and described such VA staff as “rude,” “unprofessional,” “unhelpful,” “insensitive,” and “disrespectful” (p. 230). The committee stated that VA mental health providers acknowledged that the lack of respect from frontline staff was turning off MWs to treatment or causing them to come into sessions upset. Even more concerning was the issue voiced by VA clinical leadership: the concern of having no authority over their frontline staff. We recommend that the VA should standardize evaluation procedures for frontline staff, as well as ensure that local leadership not only holds authority over frontline staff but is also held accountable for customer service that frontline staff provides.

Another improvement addresses the lack of MWs who serve as VA mental health providers; the underrepresentation of MWs among providers may at least partially explain why only 61% of MWs reported being at least ‘somewhat satisfied’ with their mental health care (Committee to Evaluate the Department of Veterans Affairs Mental Health Services, 2018). MWs often prefer to receive treatment from providers with military experience so that they do not “waste time explaining military basics to the person who was supposed to be able to help them with issues derived from that military experience” (p. 228). The committee reported that MWs were likely to leave therapy feeling offended when non-MW providers failed to understand the psychological and emotional difficulties of their military experiences. The MWs who were satisfied with the mental health care they received from a non-MW reported their provider was able to overcome the military-civilian divide by being informed of military culture, respecting their preference for certain treatments, caring about them, and demonstrating humility. Johnson et al. (2018) echo these results, finding that 95% of MWs in their study at least partly agreed (77% “agree” and 18% “partly agree”) that they would prefer a MW psychologist because of the perceived ability of MW psychologists to understand the difficulties that they were going through.

We conducted a review of the MW status for employees at a VA medical center in a metropolitan area and found that none of the psychologists (*n* = 24) providing services to MWs had ever served in the military or completed an evidence-based Veteran cultural competence training program. There was also an absence of recruiting initiatives to hire MWs into professional or leadership roles at the medical center. As highlighted above, being a MW is not a pre-requisite for providing effective care for MWs on its own. However, this raises questions about the extent to which non-MW providers (or the non-MW interns/residents that they supervise) are truly able to understand and effectively treat MWs.

The US Department of Veterans Affairs and US Department of Defense (2017) published clinical practice guidelines for the management of PTSD and acute stress disorder that encourages clinicians to use MW-centered and shared-decision making approaches that prioritize the MW’s capabilities, needs, goals, and preferences. The findings of Harik et al. (2016) support such guidelines; they found that individuals with symptoms of PTSD want to be involved in decisions regarding their care. It is concerning that the VHA is not able to adhere to these guidelines specific to MWs’ overwhelming preference to receive treatment from a fellow MW.

As a result, the military-civilian divide that MWs face in the workplace may further be exacerbated through their experience in the VA. To address this dilemma, we recommend the VA evaluate its internal promotion policies and increase recruiting efforts, hiring initiatives, and scholarship programs for MWs in graduate schools, such as the DoD’s Health Professions Scholarship Program (HPSP) for mental health providers (US Navy, 2019). The VA already has a HPSP program, but there are no current postings for mental health providers (US Department of Veterans Affairs, 2020a). We contend that an increase of MW providers, in addition to MW leadership, within the VA would help facilitate the provision of MW-centered care and implementation of the recommendations listed in this article.

Additionally, we recommend significantly expanded efforts to implement evidence-based Veteran culture competence training for all non-MW providers (as outlined in Geraci, 2019). It is probable that improvements in these areas would benefit MWs who already seek VA mental health services and attract eligible MWs who need mental health services but are not seeking them.

### Local Government

Modern warriors have unique and diverse needs, and very few of the individual community organizations are capable of wholly addressing these needs (Castro et al., 2015). Currently, there is minimal synchronization among stakeholders, no central agency responsible for vetting entities’ effectiveness, and no agency holding entities accountable for service quality (US Department of Veterans Affairs, 2017). Local (city, county and state) governments can fill a sizable portion of this organizational gap by leading in synchronization of resources and opportunities available to MWs, creating a spirit of collaboration across stakeholders and providing funding opportunities that support local initiatives. Local governments are in an optimal position to implement a strategy that extends “downstream” from the separation date to ensure successful MW reintegration and serve as an enduring hub for coordinating care services and resources (US Department of Veterans Affairs, 2017). During Phase Two, the local governments could allow for a ‘no wrong door’ approach with MWs accessing assistance from vetted stakeholders and thereby create an opportunity for MWs to customize their reintegration roadmap through ready and timely access to local resources. By helping to leverage the CVEBs, DoD efforts (e.g., SFL), VSO services, and the capabilities of the digital platforms to seamlessly weave together the services offered to MWs, the local governments can become the critical epicenter and lead transformative change.

The Governor’s (and Mayor’s) Challenges to Prevent Suicide Among Service Members, Veterans, and their Families (Substance Abuse and Mental Health Services Administration [SAMHSA], 2020) are poised to assist the local governments in leading this change through helping them develop and execute action plans that maximize the collective impact of local services, stakeholders, and federal/state/municipal agencies. The challenges have already been initiated in 27 states and 22 cities. The local teams participating in the challenges consist of a cross-section of military and civilian community leaders and aims to implement promising, best, and evidence-based practices to prevent and reduce MW suicide at the local level. It helps to advance the VA’s public health approach to suicide prevention and to implement evidence-based practices identified by the Center for Disease Control (Stone et al., 2017) into state-wide suicide prevention plans.

The ETS Sponsorship program is working closely with the Governor’s challenge teams within New York and Texas to further expand the program in both states. Within Texas, the program aligns with the state’s short-term action plan goal to prevent MW suicides by providing peer-to-peer service coordination, including training, certification, recertification, and continuing education for peer coordinators (Texas Health and Human Services Commission, 2019). This work is also nesting well with initiatives within the Texas Workforce Commission (2020) as it helps to weave together the numerous services across the state into a consolidated Texas Veteran Network, which will make it much easier for ETS Sponsors to connect reintegrating MWs to the breadth of resources offered by the state (e.g., housing, employment, education, benefits, medical, community engagement).

### Veteran Service Organizations

Typing “Veteran” into the US Internal Revenue Service (IRS) database returns a listing of 27,763 non-profit organizations with a MW related mission (US Internal Revenue Service, 2020). Some of these organizations fulfill needs unmet through local and federal government benefits. Others provide assistance in receiving VA benefits and entitlements. With approximately 245,000 MWs exiting the military each year (US Department of Veterans Affairs, 2018c), the local resources these organizations provide are critical to MWs and their families during reintegration. With many MWs not seeking care through the VA or being ineligible for VA services, there is a gap in health services, leaving ample room for these organizations. With so many options, however, it can be challenging for MWs to filter and select organizations. Selecting a poor fit is a serious concern, because a bad experience with one resource may cause the MW, already in a state of elevated stress during their reintegration, to grow frustrated and cease all assistance seeking. Increasing information about resources accessible to MWs during the selection process and improving the overall quality of resources through feedback and information exchanges is critical to improving MW reintegration. One exemplar is Pathfinder.vet - a Veteran-owned website using artificial intelligence to match MWs to appropriate resources based on reviewed experiences.

Because no single entity can address all MW needs, community-based digital platforms can play a critical role in resolving some of these issues within local communities by increasing interoperability, collaboration and accountability of organizations. For example, AmericaServes is a national service network across the nation in 17 locations and serves as a one-stop shop for services geared toward MWs that attempts to streamline services (e.g., housing, employment, VA benefits, legal assistance, and healthcare) available to reintegrating MWs. AmericaServes has received over 77,600 requests for services that were fulfilled by a network of 1,053 vetted public, private, and non-profit service organizations that meet a variety of reintegration needs (Syracuse University IVMF, 2020). America’s Warriors Partnership (AWP) (2020) and Combined Arms (2020) are other digital platform that enable best-in-class organizations to provide critical reintegration resources to empower MWs and enable them to fulfill reintegration need within Phase Two.

Another critical element to best facilitate MW reintegration is peer support or mentorship, similar to Athena/Mentor providing tangible support and encouragement to Odysseus in the Return stage. Mentors can integrate task-oriented leadership behaviors (e.g., establishing and accomplishing reintegration goals related to MWs’ unique needs) and relational-oriented behaviors (e.g., establish trusting and caring relationships with MWs), similar to helpers within the military, to reduce reintegration stress, mitigate psychological distress, reduce suicide risk, and support MWs in achieving self-actualization needs (Geraci et al., 2011; Geraci, 2018). For non-MWs with psychological disorders, the addition of peer support improves retention in active treatment, physical activity, and perceived ability to manage psychological disorders (Solomon, 2004; Cook, 2011). In 2012, the White House issued an Executive Order resulting in the VA hiring and integrating 800 peer specialists into VA mental health care, signaling the growing importance of peer-to-peer models for reintegrating MWs (Executive Order, 2012).

The ETS Sponsorship program is one example of a non-profit organization that can help to provide this critical peer support. Given the increased risk for suicide among women MWs, the ETS Sponsorship program (Geraci et al., in press) has found it be helpful to over-recruit women ETS Sponsors so that at least 30% of its sponsors are womens (compared to 15% of active US Army Soldiers being a woman, US Department of Defense, 2018). When used in conjunction with experiential data (e.g., Pathfinder.vet), full-scope referral, and community-based digital platforms, individual mentor services can help to fulfill Phase Two reintegration needs.

### Employers

Modern warriors are a valuable talent pipeline for the civilian workplace. Along with leadership experience and attention to detail, MWs bring a range of marketable traits developed in the military, including loyalty, integrity, accountability, ability to work in teams, self-reliance, perseverance under stress, safety and risk management, awareness of diversity and inclusion, and advanced technical skills (Hardison et al., 2017). Some companies, including USAA, Verizon, CSX, GE, AT&T, Capital One, Starbucks and PepsiCo, have tapped into this talent-rich pool by hiring over 117,000 MWs and spouses in recent years (Military Friendly, 2014). However, there remains a disconnect between MWs’ ability to translate skills to civilian employers and adapt to corporate culture, and civilian employers’ ability to recognize these skills as assets and adapt corporate culture to enable MWs to thrive within their new ‘tribe’ (Junger, 2016; Carter et al., 2017).

The efforts of employers to hire and retain MWs may have large effects on the high unemployment and attrition rates reported previously. Given the impact of employment upon MWs’ to reintegration and satisfying each of Maslow’s needs (physiological- salary, rent, food; love and belongingness- connecting with fellow employees; esteem- feeling competent at their trade, and self-actualization- fulfillment and reaching full potential), employer-specific recommendations are critical.

For employers to have an impactful MW program, the Chief Executive Officer (CEO) and organizational leadership must see the importance of and genuinely commit to recruiting, hiring, retaining and advancing MWs, because organizational support is more likely to occur when leaders advocate for an effort (Kotter, 2012). Beyond hiring initiatives, efforts must be synchronized with a strategy and resourced with a requisite team and funding, because initiatives that are not properly resourced often fail quickly. While increasing Veteran cultural competence of managers that supervise MWs is important, increasing cultural competence of individuals on this team is also critical (Geraci, 2019). There should also be an energetic, employee-led affinity group (consisting of MWs, family members and non-MW advocates) that extends its hand to assimilate newly hired MWs and family members. The MW affinity group should be employee-led (both MWs and non-MWs) to provide an opportunity for all employees to demonstrate their support for assisting MWs by functioning as ‘helpers’ (e.g., by taking MWs to lunch, answering questions about corporate norms and customs, dedicating volunteer time together with MWs in the community, connecting MWs to resources available in the community, advocating for MWs behind the scenes, and creating a safe space to talk).

As one of many positive examples, Howard Schultz implemented an initiative in 2013 to hire and integrate at least 10,000 MWs and their spouses into Starbucks before 2018 - a goal they exceeded. As described by MWs who work in Starbucks, Schultz not only launched the initiative but inspired the company to become a leading corporate MW advocate (Starbucks Armed Forces Network, 2017). Additionally, Starbucks established a Veteran’s and Military Affairs team, with senior leadership, to operationalize the company’s commitment beyond hiring efforts. They have dedicated over 50 Military Family Starbucks stores, provided education opportunities, made special MW aprons to recognize military service, and regularly advocated for MW and spouse employees. Lastly, Starbucks established its Armed Forces Network (AFN) consisting of MWs, spouses, and advocates, which provides an additional layer of support for MWs, spouses, and any Starbucks partner (employee) wanting to advocate for those who have served the country. Finally, every MW and spouse hired by Starbucks, since 2013, has a military-style dog tag with their name on it hanging in the executive hallway of Starbuck’s headquarters as a visible sign of the company’s commitment to MW partners.

### Colleges

The number of MWs seeking higher education on college campuses is significantly increasing (US Department of Veterans Affairs, 2018d). Many of the above employer recommendations are also applicable to college faculty and staff. Leadership should commit to recruiting, enrolling, and graduating MWs; develop a strategy that is adequately resourced; create a student-led affinity group to assist the assimilation of student MWs; synchronize internal resources to assist student MWs; collaborate with external resources, such as the VA’s VITAL program; and expand evidence-based Veteran cultural competence training for faculty and staff. Without appreciating the critical role they play in assisting MWs in their reintegration back into the community, colleges may feel ill-prepared for the influx of student MWs. However, colleges are in an optimal position to nurture MWs’ development. Through educational curriculum and leadership experiences, MWs can hone their wisdom and values developed through military service and satisfy esteem and self-fulfillment needs after graduation through continued service to their communities.

## Conclusion

Unfortunately, the increased efforts of the VA and DoD to understand and mitigate suicide for MWs appear to have had little impact on MW mental health and reducing rates of suicide, especially among younger MWs. The recommendations provided in this article are based on real-life experiences and an in-depth understanding of the shortfalls within the current research and systems related to MW reintegration and suicide. Despite the imperative need for greater knowledge about how different aspects of MW reintegration may influence long-term adjustment and suicide risk, at present, most of the research on MW reintegration is limited (Mobbs and Bonanno, 2018) and our ability to effectively support MWs and their families is hamstrung by this lack of understanding (US Department of Veterans Affairs, 2017). The work of Vogt et al. (2018) and The Veterans Metrics Initiative is a good start. But, the lack of theoretical frameworks and valid empirical data to precisely identify salient factors before, during, and after reintegration has limited the success of reintegration programs and initiatives. By no means do we contend that the recommendations that we propose in this article are exhaustive. Nonetheless, they are a consolidation of novel approaches, developed and provided by post-9/11 MWs that have potential to inform a field that lacks many salient interventions. Our goal is for the creation of a reintegration process and system for MWs that fulfills their needs positions them to continue their next ‘mission’ of serving and improving society with a newly forged MW identity, wisdom, and sense of purpose.

## Author’s Note

JG, MM, NA, RP, ED, CL, DK, and LS are all Post-9/11 Modern Warriors as per the definition in this article.

## Disclosure

The views expressed in this article are those of the authors and do not necessarily reflect the position or policy of the Department of Veterans Affairs, Department of the Army, affiliated organizations or the United States government. The authors did not receive any specific grant from funding agencies in the public, commercial, or not-for-profit sectors to write this article.

## Data Availability Statement

The original contributions presented in the study are included in the article/supplementary material, further inquiries can be directed to the corresponding author/s.

## Author Contributions

JG: primary author, coordinated participation of other authors, conducted final editing, and developed manuscript theoretical concept and structure. MM: subject matter expert for reintegration stressors and wrote portions on this topic. EE: postdoctoral researcher on Veteran suicide, and guided manuscript organization and editing. BD: subject matter expert for Greek mythology and wrote portions related to Odysseus’s journey. NA: subject matter expert for employment and wrote portions on recommendations for private organizations. RP: key orchestrator of Starbucks’ Veteran initiatives and wrote portions on employment. ED: subject matter expert for Veteran Service Organization (VSOs) involvement in Modern Warrior reintegration and wrote portions on VSOs. CL: subject matter expert related to the US Army and wrote portions on the US Army. DK: research assistant with Geraci, conducted editing of the manuscript, created references, and formatted tables. JJ: research assistant with Geraci, conducted editing of the manuscript, created references, and formatted tables. GC: subject matter expert for employment and wrote recommendations for private organizations. LS: subject matter expert for local government and wrote portions on local government recommendations. YS: postdoctoral researcher on Veteran suicide and contributed to editing of the manuscript. MG: subject matter expert for VA recommendations and wrote portions on the VA. All authors contributed to the article and approved the submitted version.

## Conflict of Interest

The authors declare that the research was conducted in the absence of any commercial or financial relationships that could be construed as a potential conflict of interest.

## Footnotes

1 Notably, MWs who do not experience this second rite of passage are still considered MWs and benefit from the training and development received in the military. Many of these MWs desire to engage the enemy to demonstrate their worth to their unit and comrades.
